# The Israeli strain IS-98-ST1 of West Nile virus as viral model for West Nile encephalitis in the Old World

**DOI:** 10.1186/1743-422X-1-9

**Published:** 2004-11-18

**Authors:** Marianne Lucas, Marie-Pascale Frenkiel, Tomoji Mashimo, Jean-Louis Guénet, Vincent Deubel, Philippe Desprès, Pierre-Emmanuel Ceccaldi

**Affiliations:** 1 Unité des Interactions Moléculaires Flavivirus-Hôtes, Institut Pasteur, Paris, France; 2 Unité de Génétique des Mammifères, Institut Pasteur, Paris, France; 3 Institute of Laboratory Animals, Kyoto University Graduate School of Medicine, Kyoto, Japan; 4 Unité de Biologie des Infections Virales Emergentes, Institut Pasteur, Lyon, France; 5 Institut Pasteur of Shangai, Shangai, P.R. China; 6 Département de Virologie, Institut Pasteur, Paris, France; 7 Unité Epidémiologie et Physiopathologie des Virus Oncogènes, Institut Pasteur, Paris, France

## Abstract

West Nile virus (WNV) recently became a major public health concern in North America, the Middle East, and Europe. In contrast with the investigations of the North-American isolates, the neurovirulence properties of Middle-Eastern strains of WNV have not been extensively characterized. Israeli WNV strain IS-98-ST1 that has been isolated from a white stork in 1998, was found to be highly neuroinvasive in adult C57BL/6 mice. Strain IS-98-ST1 infects primary neuronal cells from mouse cortex, causing neuronal death. These results demonstrate that Israeli strain IS-98-ST1 provides a suitable viral model for WNV-induced disease associated with recent WNV outbreaks in the Old World.

## 

West Nile virus (WNV) is a single-stranded RNA flavivirus (family *Flaviviridae*, genus flavivirus) with a worldwide distribution ranging Africa, Europe, the Middle East, and Asia. WNV was first recognized in the Western Hemisphere in 1999. The emergence of WNV has been associated with a dramatic increase in severity of disease in humans and other species[1,2]. Recent WNV epidemics which include meningitis, encephalitis and poliomyelitis-like syndrome in humans have been reported in Europe, the Middle-East and in North America. During the summers of 2002 and 2003, more of 13,000 human cases and 500 deaths were reported from the United States, drawing the attention of WNV illness as an important public health concern.

Comparison of WNV strains identified two major genetic subtypes: the lineage II (enzootic strains from tropical Africa and Madagascar island) and the lineage I (tropical african strains) that caused the outbreaks of WNV infection in North Africa, Europe, Israel, and in the United States. Nucleotide sequencing revealed that American strains of WNV isolated between 1999 and 2000 are nearly identical to Israeli strains of WNV isolated in 1998 and 2000 [3,4]. This close relationship could be explained by the fact that an Israeli WNV strain was introduced in New York City in 1999 [4].

The murine model of WNV-associated encephalitis has been widely used to address the viral pathogenesis[5]. Strains of WNV isolated in the United States were found to be highly neuroinvasive in adult mice following intraperitoneal (i.p.) inoculation[6]. In contrast of the investigations of the North-American WNV strains, the virulence phenotype of Israeli strains of WNV has not been extensively characterized. The WNV strain IS-98-ST1 has been isolated from cerebellum of a white stork during an outbreak in Israel in 1998[7]; its phenotypic characterization was performed after 3 passages in the mosquito cell line *Aedes pseudoscutellaris *AP61[8] and its complete genomic sequence determined (GenBank accession number AF481864). Virus titration was performed on AP61 cells by focus immunodetection assay as previously described [9]. Infectivity titers were expressed as focus forming units (FFU).

In this study, we demonstrated that IS-98-ST1 has a high neuroinvasive potential in adult C57Bl/6 mice, and that the virus is capable to replicate in primary neuronal cultures from mouse brain cortex.

Mouse experiments were performed according to the European Convention 2001–486. After anesthesia, six-week-old female C57BL/6 mice (Harlan, France) were inoculated with 1,000 FFU of WNV via different routes (15 animals per group): intraperitoneal (i.p.), intradermal (i.d.), intracerebral (i.c.), and intranasal (i.n.). At Days 5 and 7 of infection, three animals per group were euthanasied; brain and spinal cord were rapidly removed, processed for viral titration or sectioned on cryostat (Jung Frigocut; 14 μm thick sections). Sections were fixed with 3.7% formaldehyde or acetone for 30 min and processed for indirect immunofluorescence with mouse polyclonal anti-WNV antibodies[8]. Some sections were also processed for Glial Fibrillary Acidic Protein (GFAP) using a rabbit polyclonal antibody (Promega). Sections were further washed, mounted and observed with a fluorescence microscope (DMRB Leica).

When infected i.c., mice died at day 7.3 ± 1 post-infection (p.i.) ; 100% mortality was also reached after i.p., i.n., or i.d. inoculation but with delayed kinetics (day 9.5 ± 0.5, 10.7 ± 0.7 and 10.5 ± 0.5 p.i. respectively). In all cases, WNV-infected mice exhibited characteristic disease progression with hind limb paralysis, cachexia and tremors. By day 7 p.i., WNV was found in brain tissue in all mice, reaching virus titers from 3.10^5 ^(i.d. route) to 3.10^8 ^FFU/g (i.c. route).

To investigate WNV location within the CNS, cryostat brain sections from three WNV-infected mice were assessed for the presence of viral antigens by immunofluorescence at day 7 p.i. When inoculated i.c, virus was found widespread in most of the brain structures (whereas no signal was seen in mock-infected controls), including cortex (Fig. 1A), pyramidal neurons of the hippocampus (Fig. 1B), spinal cord and olfactory bulb. In contrast, a lower level of infection was observed after i.p., i.d. or i.n. inoculation (Fig. 1E), showing regional variations according to the route of inoculation (Fig. 1C and 1D). In all sections, WNV-infected cells were negative for GFAP (Fig 1F). This suggests that neurons are the principle targets of infection in the CNS.

**Figure 1:**
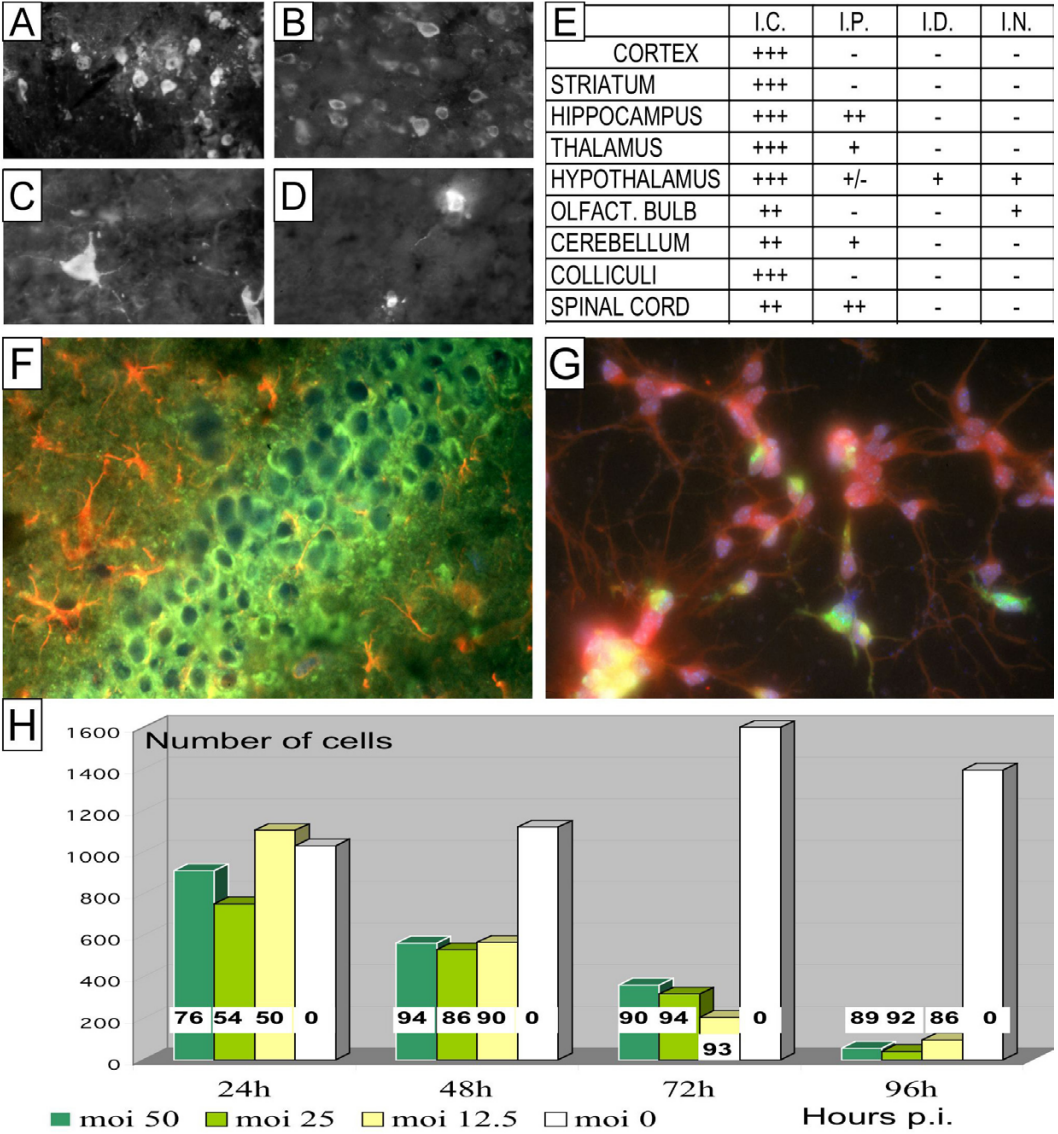
**A **to **F**: WNV antigens in different regions of the mouse CNS. Mice were inoculated with 10^3 ^FFU of IS-98-ST1 WNV upon different routes (i.c., i.p., i.n., i.d.); at Day 7 of infection, mice were euthanazied, brains were cut in 14 μm thick cryostat sections, and processed for immunofluorescence using anti-WNV serum (obtained from i.p.-inoculated resistant mice) as primary antibody. **A**: hippocampus (pyramidal layer), i.c. inoculation. **B**: frontal cortex, i.c. inoculation. **C**: spinal cord, i.p. inoculation. **D**: olfactory bulb, i.n. inoculation. Magnification: × 350. **E**: Average levels of infection of the different brain structures was estimated on 10 different sections for each of the 3 animals per group (I.C.: intracerebral, I.P.: intraperitoneal, I.D.: intradermal; I.N.: intranasal) according to the scale: +++: more than 10 positive cells per microscopic field; ++: between 3 and 9 positive cells; +: 1 or 2 positive cells; -: no positive cell. **F**: Immunodetection of WNV antigens (green) and Glial Fibrillary Acidic Protein (red) in cryostat section of WNV-infected mouse brain, day 7 of infection, i.c Magnification: × 700. **G, H**: WNV infection in primary neural cultures from C57BL/6 mouse brain cortex. Primary cultures were performed as described in text and infected with IS-98-ST1 WNV. **G**: Detection of WNV antigens (using anti-WNV mouse immune serum and a FITC-conjugated secondary antibody, green staining) and neuronal specific enolase (using a rabbit polyclonal antiserum and an anti-rabbit polyclonal antibody made in goat conjugated with Texas Red, red staining) by immunofluorescence at 24 h p.i. (m.o.i. 12.5). Magnification: × 700. **H**: Kinetics of infection and variation of cell number at various times post-infection for different m.o.i; three cultures for each m.o.i. were fixed and processed for WNV antigen detection by immunofluorescence, whereas cell nuclei were visualized with DAPI. Cell nuclei of adherent cells were counted in 8 different different fields for the three cultures (histogram) whereas the percentage of infected cell was estimated by counting WNV antigen positive cells and cell nuclei; the percentage of infected cells is indicated as values (%) in white squares.

For ex-vivo experiments, primary neuronal cultures were prepared from the brain cortex of C57/BL6 mouse embryos (day E15) (Harlan, France)[10]. Briefly, after rapid removal of the embryos and dissection of brain cortex, mechanical dissociation and centrifugation were performed; the cells were seeded on slides and grown in NeuroBasal/B27 medium (Invitrogen Corporation) and, around 10 days after plating, were infected with WNV at different multiplicities of infection (m.o.i.). Cell cultures were constituted by more that 90% neurons, as assessed by immunocytochemistry. At different times post-infection, cell culture supernatants were processed for viral titration; cells were fixed and processed for immunofluorescence detection for viral antigens (see above) or neural cell typing, using either an anti-neuron specific enolase (NSE) (Zymed) or an anti-GFAP (Promega). After 24 h of infection at a m.o.i of 25, ~50% of cells were infected (Fig. 1H). By 40 h p.i., 90% of cells became infected and > 10^7 ^FFU of WNV per ml was detected in the culture supernatant. Time course studies showed that IS-98-ST1 infection induced cell death through neuronal necrosis within 48 h of infection, and ~90% of cells had detached by 96 h (Fig. 1H). Whatever the time of infection, only neuronal cells were permissive for IS-98-ST1 as judged by double immunofluorescence staining for WNV antigens and NSE (Fig. 1G). GFAP positive cells, i.e. astrocytes, that constitute less than 10% of cells appeared to be relatively resistant ot WNV infection. To confirm this, astrocyte-enriched primary cultures from the brain cortex of mouse embryos were infected with IS-98-ST1 at a m.o.i of 50. By 48 h p.i., only 5% of GFAP immunoreactive cells expressed viral antigens (data not shown).

Although our study was limited in its scope, the results indicate that WNV strain IS-98-ST1 is suitable as viral model for West Nile encephalitis in the Old World. The Israeli strain IS-98-ST1 that caused the epizootic in Israel in 1998, was found to be highly neuroinvasive in mice following peripheral inoculation. Consistent with this observation, we reported that IS-98-ST1 has an i.p. LD50 value as low as 10 FFU[8]. IS-98-ST1 infection has allowed us to determine the role of the type-I interferon (IFN) response in controlling WNV infection and that IFN-inducible OligoAdenylate Synthetase molecules may play an important role in the innate defense mechanism against WNV[8,11]. High viral titers could be recovered in mouse brains whatever the route of inoculation (i.c., i.p., i.d., i.n.). Viral antigens were detected in most brain structures at day 7 of infection, consistent with the notion that IS-98-ST1 is able to reach the CNS and then replicate in the brain. Infected C57Bl/6 mice showed neurological symptoms and lethality, confirming the high neurovirulent characteristics of IS-98-ST1, that were described in another susceptible mouse model of WNV (North-American strain) infection [12]. These features may be linked to the predominance of neurological symptoms that have been observed in hospitalized patients during Israeli outbreaks [13] or during natural infections of horses [14]. Our data are compatible with a previous report[15] indicating that WNV replicates locally in draining lymph nodes in mice inoculated subcutaneously, then in the spleen and in multiple sites in the CNS, although the sites of extraneural viral infection and the possible cells that could be involved in such a passage remain elusive. The dissemination of foci of infection within the brain that is observed in our study is compatible with virus passage through the blood-brain barrier. However, the fact that infected neural cells are detected in the olfactory bulb after intra-nasal inoculation suggests that an intraneural transport of WNV cannot be ruled out. Such neuroinvasive properties have also been reported for WNV variants from North America in experimental infection in rodents [16] and avian species as well as in natural infections in horses or birds[5,17]. Although some of these studies support the infection of neural cells by WNV within the CNS, none used double immunocytochemistry for WNV antigen and cell typing.

Our study confirms the neurotropism of WNV and the huge preferential infection of neurons *in vivo*. Because neurons are believed to be main target neural cells of WNV, we developed an *ex-vivo *model of infection, by culturing primary neural cells from the brain cortex of susceptible mice. More than 90% of the neurons are found to be infected by IS-98-ST1 and infected neurons undergo necrosis. In contrast, astrocytes were mainly resistant to WNV infection. This is consistent with *in vivo *data showing a massive infection of brain structures such as brain stem, hippocampus and cortex of WNV-infected animals [12] and human patients[5]. The high neuropathogenicity of IS-98-ST1 isolated from a stork in Israel in 1998, as well as WNV strains present in North America does contrast with the low pathogenicity of most ancestral strains of WNV[18,19]. In conclusion, the Israeli strain IS-98-ST1 of WNV provides a relevant model for assessing the identification of viral factors that may responsible for West Nile pathogenesis.

## Authors' contribution

ML carried out *ex-vivo *studies, M-PF and TM participated in *in vivo *experiments, VD and J-LG revised critically the article, PD and P-EC have written, drafted the article, and participated to in vivo and ex-vivo experiments.

## Competing Interests

The authors declare that they have no competing interests.
